# Acute Kidney Failure: When Multiple Myeloma Doesn´t Give Additional Clues

**DOI:** 10.7759/cureus.7664

**Published:** 2020-04-14

**Authors:** Ana Cerqueira, Tiago Seco, David Paiva, Helio Martins, Jorge Cotter

**Affiliations:** 1 Internal Medicine, Hospital Senhora Da Oliveira, Guimarães, PRT

**Keywords:** multiple myeloma, acute renal failure, renal biopsy

## Abstract

Multiple myeloma (MM) is characterized by a proliferation of malignant plasma cells and a subsequent overabundance of monoclonal paraprotein. This disease commonly presents with hypercalcemia, kidney failure, anemia, and bone lesions. Acute kidney failure (AKF) as an initial presentation of MM has rarely been reported. Herein, we present a case of a 49-year-old female who was admitted to our intensive care unit (ICU) for AKF in June 2017. The patient was admitted to our emergency room (ER) with abdominal pain and biliary vomiting within six days. From the laboratory tests, we highlight a serum creatinine of 19 mg/dl and urea of 377 mg/dl. The physical examination was globally unremarkable. Once clinically stable, she was admitted to our infirmary with a creatinine of 8.00 mg/dl. The patient underwent an extensive study: markers for hepatitis B and C, human immunodeficiency syndrome (HIV), and autoimmune markers were all negative; renal ultrasound, abdominal and pelvic CT had no relevant alteration; and the skeletal survey had no significant change. Peripheral blood smear showed no abnormalities. Serum immunoglobulin analysis revealed an elevated immunoglobulin A (IgA). Serum protein electrophoresis showed a monoclonal spike and urine protein electrophoresis showed an increased amount of protein consistent with Kappa light chains. The Kappa:Lambda chain ratio was increased. In order to understand the etiology of this AKF, we ended up performing a kidney biopsy, which was compatible with a myeloma kidney. The patient was transferred to the Portuguese Oncology Institute in Porto and initiated chemotherapy. Two months after the hospital discharge, creatinine levels were stable around 1.5 g/dL. This case illustrates AKF as the initial and sole presentation of MM. This presentation, even though previously reported, is very uncommon, especially considering that it occurred in a young woman and it was associated with light chain precipitation of IgA. MM is an important differential diagnosis in AKF, particularly when excluded pre and post-renal etiologies. Although being an invasive procedure with inherent possible complications, a kidney biopsy is still a very important procedure that was essential in this case to achieve a final diagnosis and, therefore, the patients' treatment.

## Introduction

Multiple myeloma (MM) is a debilitating malignancy that is part of a spectrum of diseases ranging from monoclonal gammopathy of unknown significance (MGUS) to plasma cell leukemia. First described in 1848, MM is characterized by a proliferation of malignant plasma cells and a subsequent overabundance of monoclonal paraprotein (M protein). MM accounts for 1% of all cancers and is the second most common hematologic malignancy after lymphoma [1]. The estimated worldwide five-year prevalence is approximately 230,000 patients. In the Western world, the age-standardized incidence has been reported to be approximately five cases per 100,000. The median age at diagnosis is approximately 66-70 years, with 37% of patients being younger than 65. MM is extremely rare in those less than 30 years of age with a reported frequency of 0.02% to 0.3% and appears to occur slightly more frequently in men. In general, MM is not considered to be a genetic disease, however, familial cases, albeit rare, do exist. Interestingly, it was observed that relatives of patients with MGUS as compared to normal controls had a higher relative risk of developing MGUS (2.8 fold), MM (2.9 fold), Waldenström macroglobulinemia (4.0 fold), and chronic lymphocytic leukemia [2]. This disease commonly presents with hypercalcemia, renal failure, anemia, and bone lesions (the CRAB features), which are used in diagnostic evaluation for MM. This set of symptoms consists of hypercalcemia and bone pain secondary to lytic bone lesions and increased osteoclastic activity. Anemia is found in about 73% of MM patients at presentation. Renal failure is the focus of this case report and occurs most commonly due to pathologic light chain deposition in the kidneys. Elevated creatinine is found in about half of the MM patients at presentation. While renal impairment is a frequent presenting symptom, it rarely is the singular presenting symptom of MM [3]. To determine the cause of kidney failure, we often need to perform a kidney biopsy in order to take a closer look at the glomeruli and tubules. When a patient has cast MM, it is possible to see the tubules full of proteins that block the inside of the tubule. The glomeruli of the kidney are typically not affected in the cast nephropathy and usually appear normal [4].

## Case presentation

A 49-year-old female with a past medical history of dyslipidemia and depression was admitted to our intensive care unit (ICU) for acute kidney failure. Her active medication included citalopram, lorazepam, amitryptiline, and pregabalin. None of these drugs had been recently initiated. The patient presented to our ER with unspecified abdominal pain, biliary vomiting, asthenia, and anorexia, which she was experiencing for the past six days. In the two days previous to admission, she also had the notion of decreased diuresis. From the initial ER evaluation, we highlight a creatinine of 19 mg/dl, urea of 377 mg/dl, and Hb 9.6 g/dl (normocytic and normochromic anemia). She also had gasimetry with severe metabolic acidosis with pH 7.18, partial pressure of carbon dioxide (PCO2) 25.5 mmHg, partial pressure of oxygen (PO2) 120.7 mmHg, K+ 5.08 mmol/L, Na+ 134.7 mMol/L, Lact 0.9 mmol/L, oxygen saturation (SatO2) 97%, and bicarbonate (HCO3)- 9.1 mmol/L. Urine analysis showed proteinuria and the urine sodium was 60 mEq/L. The patient had no recent history of non-steroidal anti-inflammatory drugs (NSAIDs) use, neither did she describe symptoms of infection. She was dehydrated probably in the context of vomiting. The patient presented with no other symptoms and had no other significant past medical history. Familiar and social history were noncontributory. She was pale and her mucous membranes were discolored. For the remainder, the physical examination was unremarkable.

**Figure 1:**
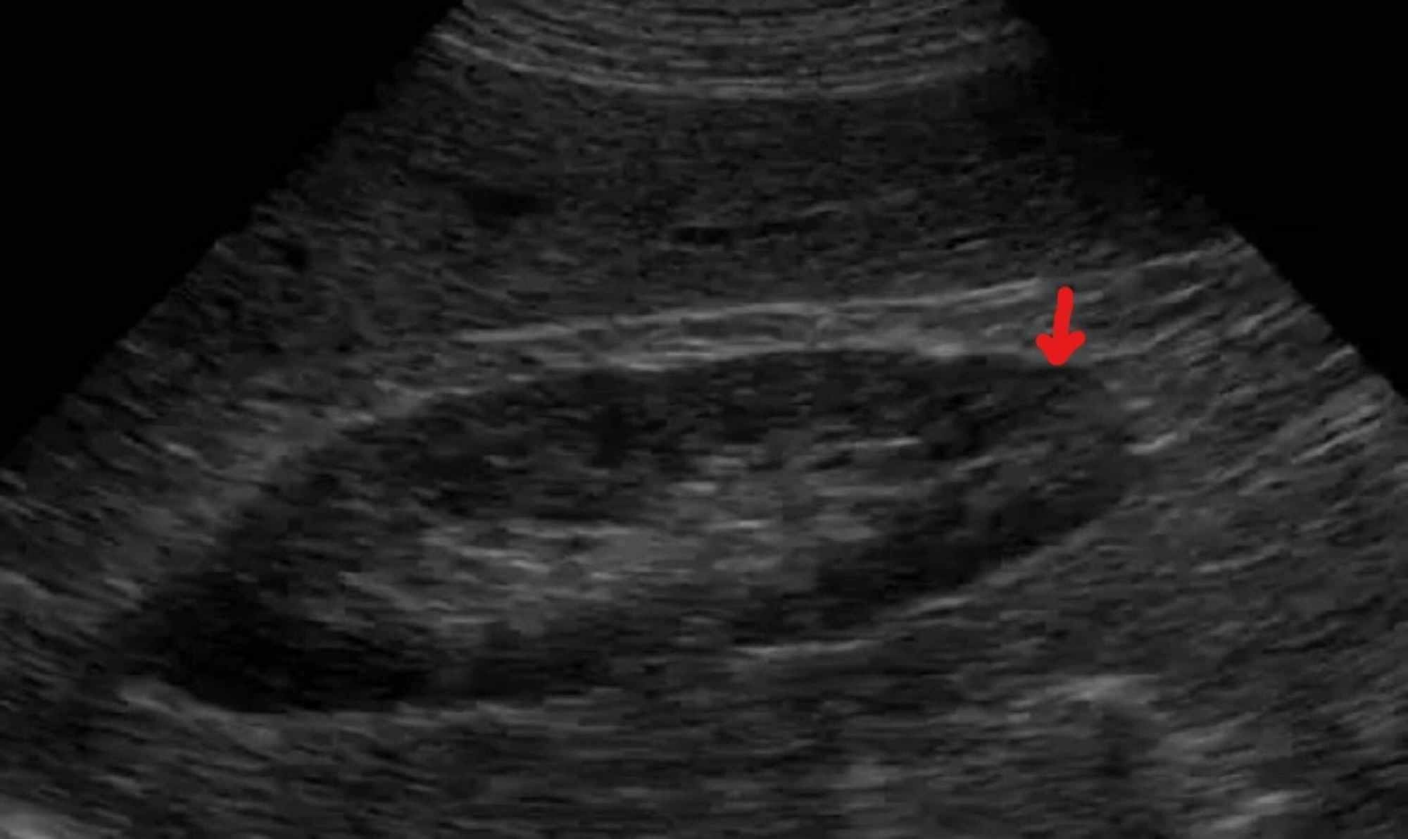
Right Kidney Ultrasound There was no mass lesion, calcification, or hydronephrosis, but a bilateral loss of parenchymal-sinus differentiation was described, likely to be related to the inflammatory process.

Baseline creatinine was obtained from the patient’s primary care physician, which was 1.32 mg/dL three months prior. The patient was diagnosed with acute kidney failure associated with severe acidosis and, at this point, she was anuric. In this context, she was admitted to the ICU. During the eight days of ICU hospitalization, the patient was volume resuscitated with intravenous fluids for decreased renal perfusion secondary to dehydration. She needed renal replacement therapy during the first six days. Once stable, she was admitted to the internal medicine infirmary. Her creatinine at admission was 8.00 mg/dl. The patient underwent extensive laboratory and imaging studies, which were all inconclusive or nondiagnostic. The comprehensive laboratory panel included hepatitis B surface antigen (HbsAg), hepatitis C antibody (HCV Ag), HIV, anti-nuclear antibody (ANA), antineutrophil cytoplasmic antibody (ANCA), and glomerular basement membrane antibody (GBM Ab), which were all negative. She did another renal ultrasound (the first one was made while she was in the ER) and showed both kidneys measuring 12 cm of bipolar diameter with no evidence of renal mass lesions, hydronephrosis, or calcifications (Figure 1).

**Figure 2:**
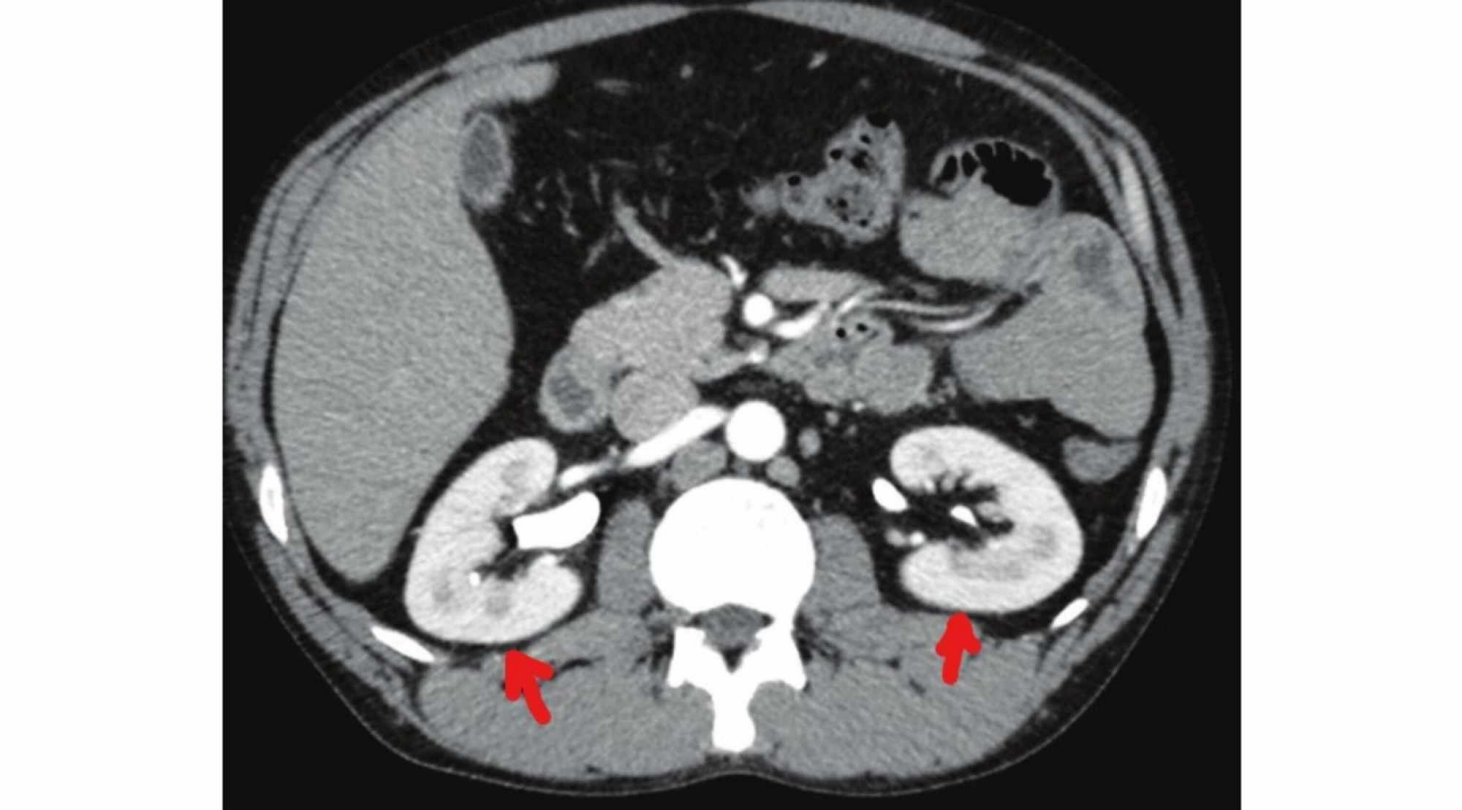
Abdominal CT Scan Study without major changes for liver, gallbladder, pancreas, and spleen, with kidneys of regular and symmetrical dimensions

Abdominal and pelvic CT were unremarkable in the present context (Figure 2).

**Figure 3:**
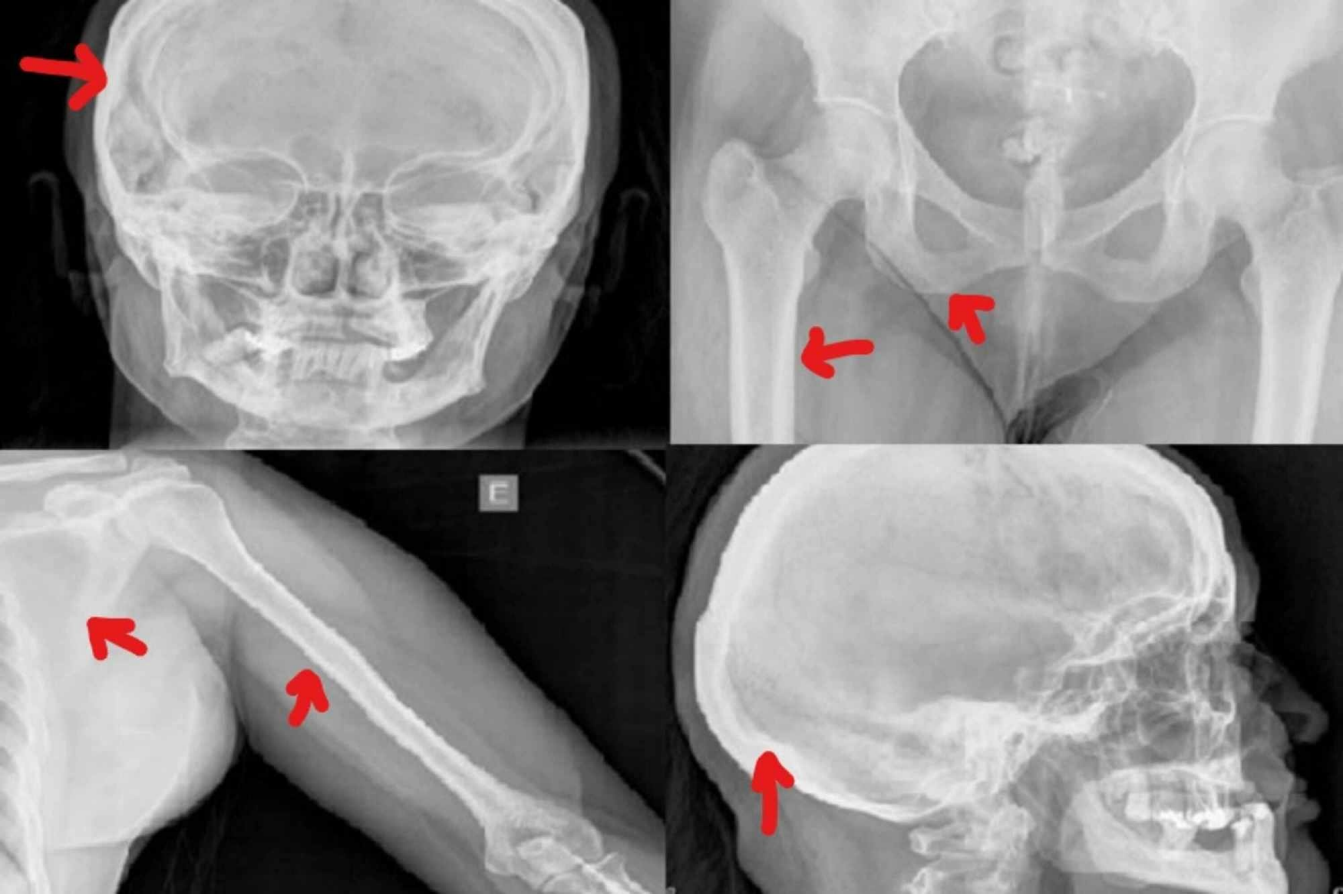
Set of Broad Bones Without Evidence of Lytic Images A) Holographic cap, B) Femur and thigh, C) Scapula, rib cage, and humerus, D) Occipital area

A skeleton survey showed no focal lytic lesions in the central skeleton or proximal appendicular skeleton (Figures 3A-3D).

**Figure 4:**
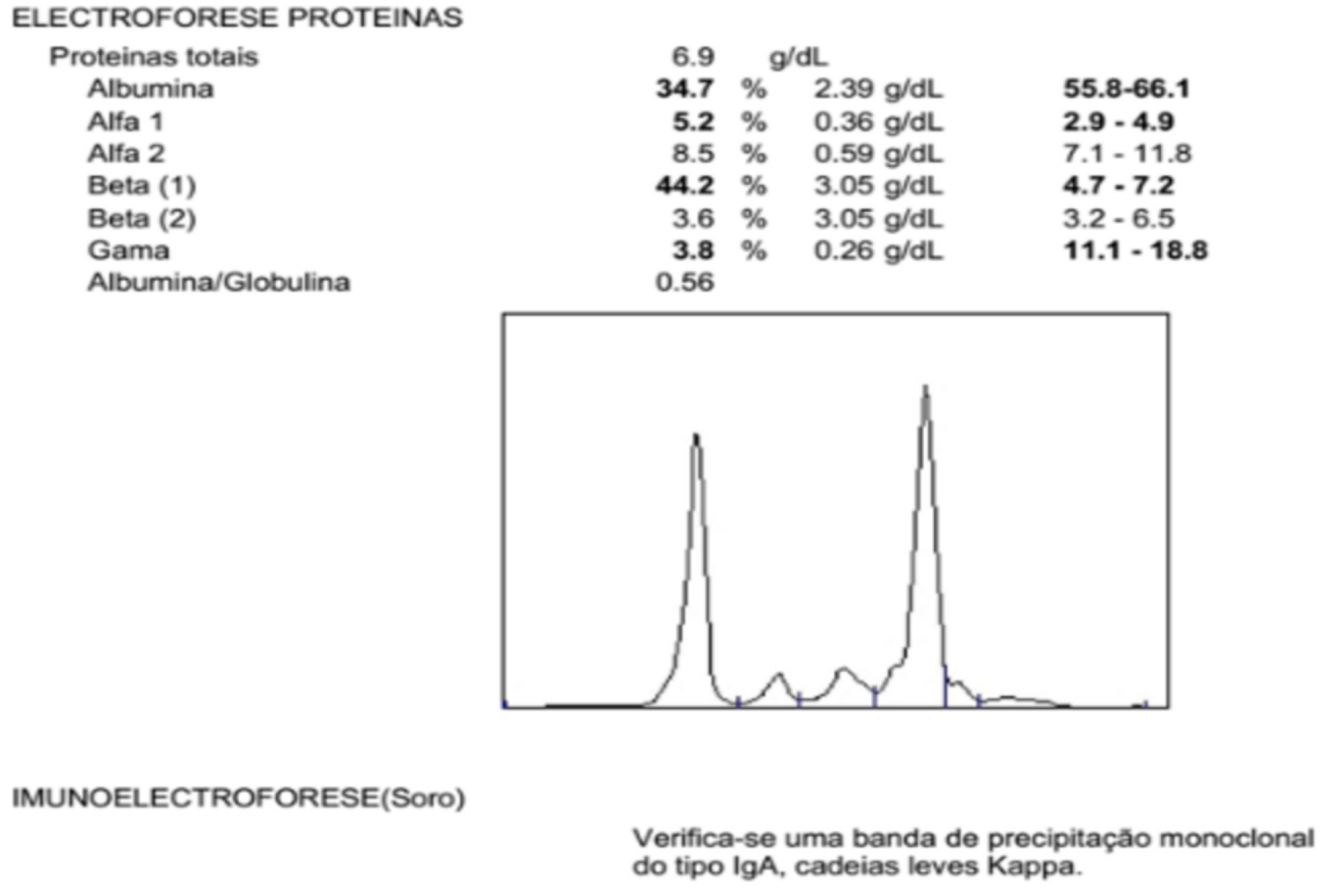
Serum Protein Electrophoresis of Our Patient

Serum immunoglobulin analysis revealed IgA 3430 mg/dl (reference values: 114-457), IgG 204 mg/dl (reference values: 793-1590) and IgM 34.4 mg/dl (reference values: 29-226). Peripheral blood smear showed no abnormalities. Serum protein electrophoresis (SPEP) was positive for a monoclonal spike (M-spike) of 44.2% in the Beta1 region (Figure 4).

**Figure 5:**
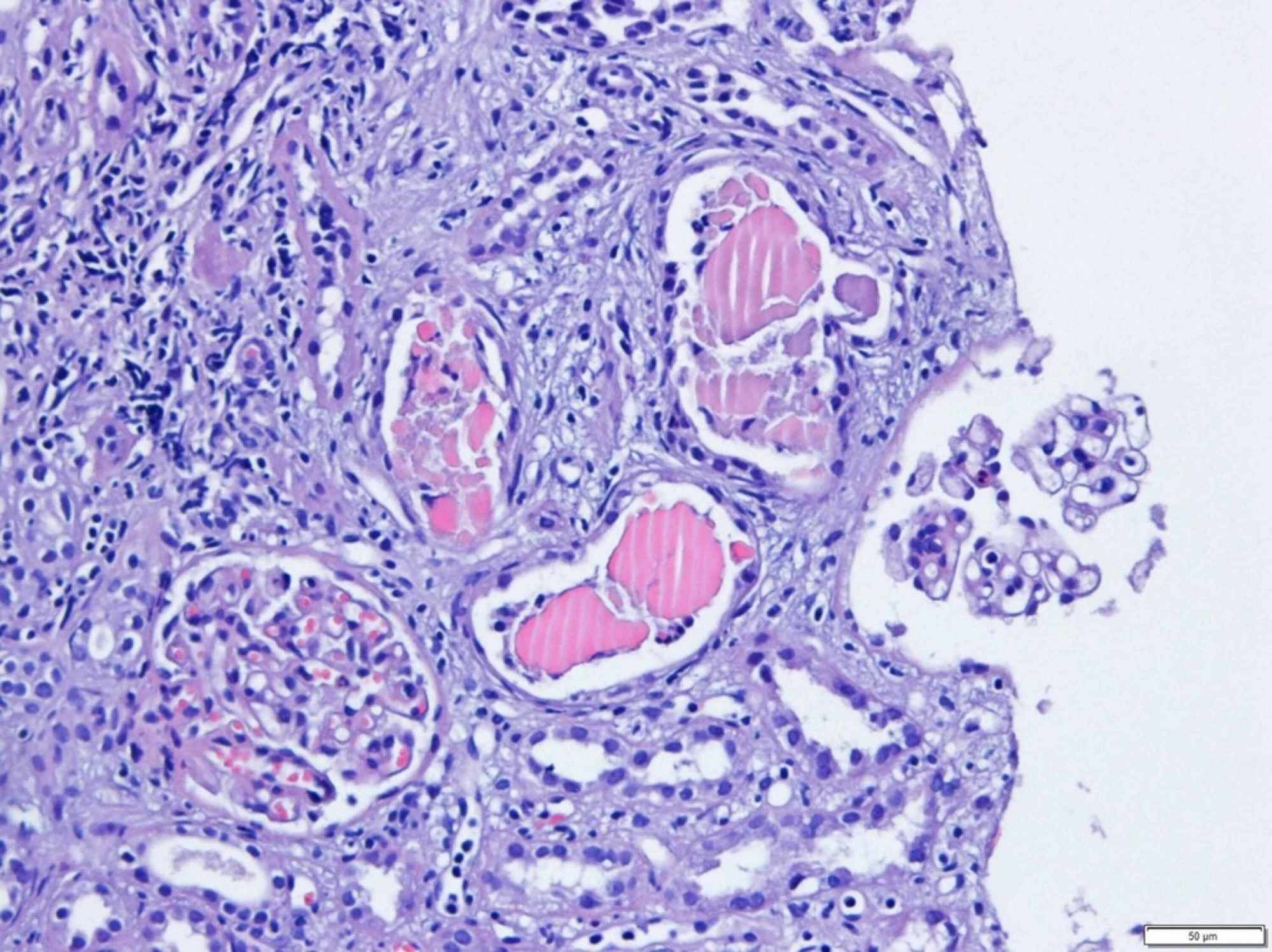
Renal Biopsy with Abundant Interstitial Fibrosis and Lymphocytic Infiltrates

Urine protein electrophoresis (UPEP) showed 1210 mg/dl of Kappa light chain proteins. Serum immunoelectrophoresis revealed a Kappa:Lambda chain ratio of 39.03 (reference values 1.35-2.70). Bone marrow biopsy was performed and in order to study the cause of this AKF, we also performed a kidney biopsy. We first accessed the kidney biopsy report that revealed tubular necrosis and numerous fractured cylinders sometimes involved by cellular elements. Interstitium with fibrosis and foci of lymphocytic and eosinophilic infiltrates suggested allergic interstitial nephritis (Figure 5).

**Figure 6:**
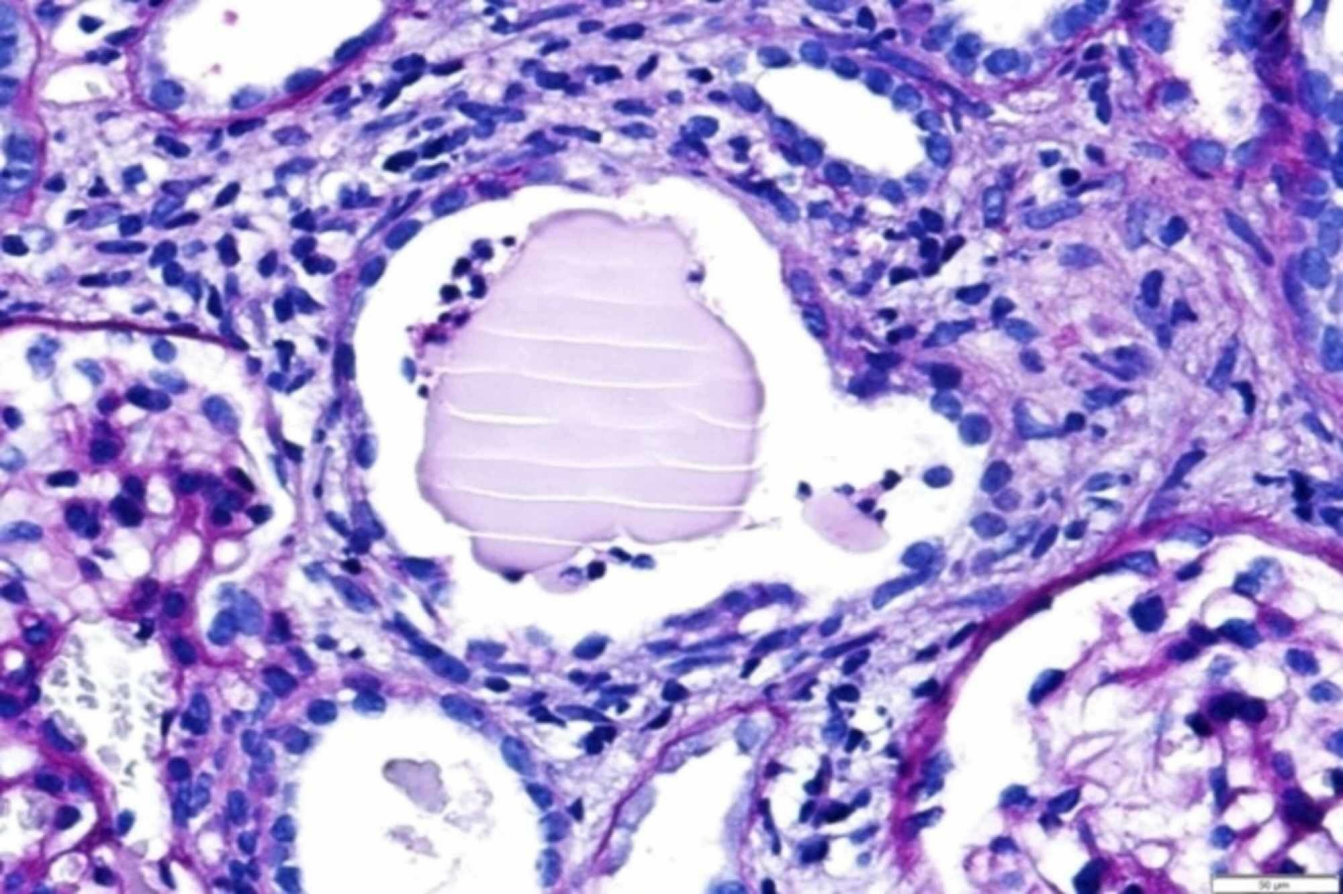
The "Foreign Body" from the Myeloma Kidney Found in the H&E Stain H&E: hematoxylin and eosin

The excretion of light chains causes tubular damage, generating obstructive nephropathy known as "myeloma kidney." This nephropathy is characterized by three main components: proximal renal tubular atrophy, the formation of eosinophilic cylinders in the distal tubule, and inflammation with interstitial fibrosis [5]. The typical histopathological finding is the presence of light chain eosinophilic cylinders inside the lumen of the renal tubules surrounded by cells - multinucleated giants generating a "foreign body" reaction in the distal tubules and collecting duct (Figure 6).

Studies of the immunofluorescence and ultrastructure of the cylinders identified Tamm-Horsfall mucoprotein, which is synthesized by distal tubular cells, validating the diagnostic suspicion of myeloma kidney. It is also important to remember that the congo red dye was made and no structure was identified. The monoclonal immunoglobulin deposition disease was also suspected (it is also congo red negative), but in this case, the most common histological finding would be glomerulosclerosis nodular mimicking a picture of diabetic kidney or membranoproliferative glomerulonephritis, which were not documented in our stains. Lastly, we had a patient with severe kidney failure and no bone pain at admission and, therefore, acquired Fanconi syndrome did not fit the explanation for this kidney injury.

The immunophenotyping study showed, a posteriori, 60% of bone marrow monoclonal plasma cells with 100% CD138 +, 100% CD38 +, and 45% CD20 +.

The patient was transferred to the Portuguese Oncology Institute in Porto and remained as an inpatient in that hospital for two more weeks. She initiated chemotherapy with bortezomib and thalidomide, associated with dexamethasone, being discharged after five cycles of treatment. At our last assessment, she had a SPEP with no M-spike and the Kappa:Lambda chain ratio was 6.73. The patient was also referred for nephrology and internal medicine consultation, and two months following discharge, the creatinine was stable between 1.3 and 2.3 g/dl.

## Discussion

Multiple myeloma accounts for 1% of all cancers and approximately 10% of all hematologic malignancies. Unlike other malignancies that metastasize to the bone, the osteolytic bone lesions in multiple myeloma exhibit no new bone formation. Bone disease is the main cause of morbidity and can be detected on routine skeletal radiographs, low‐dose whole-body computed tomography (WB‐CT), magnetic resonance imaging (MRI), or fluoro‐deoxyglucose positron emission tomography/computed tomographic scans (PET/CT). Other major clinical manifestations are anemia, hypercalcemia, kidney failure, and an increased risk of infections [6].

Despite having already been described, AKF remains an unusual form of presentation of MM. The ongoing renal failure in MM often results from tubular nephropathy because of circulating paraproteins secreted by plasm cells clones, most commonly immunoglobulins and free light chains [7].

In 2009, Prakash et al. analyzed 50 patients from Eastern India that developed renal failure preceding the diagnosis of MM. Their work showed that renal dysfunction prior to diagnosis is most frequently manifested as AKF, followed by chronic renal failure, and, lastly, nephrotic syndrome. In this study, 84% of the patients presented with renal impairment before a diagnosis of MM. The same study determined that AKF was the most common manifestation of renal dysfunction in MM. In fact, their main conclusion was that MM should be considered as a possible cause of unexplained AKF in patients older than 50 [6]. Irish AB et al. had already shown in 1997, in a study of 56 patients with MM, that renal failure was the first presentation of MM in half of them; however, a majority also presented with hypercalcemia that actually worked as a precipitant of renal failure. Though there is substantial evidence that renal failure is a common presentation of MM, there are just a few published cases where renal failure was the sole manifestation of MM. Gastelum et al., also reported a case of AKF as an initial presentation of MM, without associated hypercalcemia or anemia. Even so, this case occurred more in favor of the known statistics of the disease: male, 54 years old, with a monoclonal peak of IgG [3].

Our case is even more unusual considering that it occurred in a young woman and in association with the light chain precipitation of IgA. Upon initial laboratory testing, history, and physical exam, the differential diagnosis for this case included chronic renal failure and acute renal failure, for which many possible causes exist. Acute or sub-acute onset etiologies were most likely found in our patient given her baseline creatinine of 1.32 mg/dL three months prior with normal-sized kidneys on ultrasonogram. In determining the cause of the patient´s AKF, it was important to distinguish whether her AKF was pre-renal, intrinsic renal, or post-renal. According to her euvolemic status after the ICU hospitalization, stable vital signs, blood urea nitrogen (BUN)/creatinine ratio, urine sodium, pre-renal causes were excluded. Furthermore, the absence of obstruction or hydronephrosis on renal ultrasound excluded a post-renal cause. Thus, it was concluded that the patient´s AKF was secondary to an intrinsic renal pathologic process. Given the fact that corrected calcium at presentation was 8.7 mg/dl, and she was experiencing no bone pain, although she had a Hb of 9.6 g/dl, multiple myeloma was quite low on the differential diagnosis. Considering the patient´s history, the most common differential diagnoses were not very strong possibilities: she was not diabetic, the dehydration has been corrected and now she was euvolemic with normal urine electrolytes, and she did not have recent medication changes. Between the other causes of intrinsic AKF, tumor lysis syndrome, hemolysis, thrombotic thrombocytopenic purpura, and hemolytic uremic syndrome (TTP/HUS), contrast-induced nephropathy, and atheroembolism were all unlikely causes given the lack of risk factors and pertinent negative history of previous chemotherapy, surgery, medications, or infection. It´s important to remember in this phase that she was medicated at home with citalopram and there had been some reports of acute interstitial nephritis (AIN) related to this drug. However, this did not justify our patients' case since she had been taking citalopram for over three years. In addition, the typical histopathological data in an AIN disclose extensive inflammatory infiltrates within the interstitium that are composed of lymphocytes and macrophages. Very often, granulomas are seen on the stains of H&E. The patient with AIN has peripheral eosinophilia in more than 85% of the cases reported, which was not present in our patient's blood analysis during the entire hospitalization period [8].

Although the patient lacked bone pain, pathologic fractures, weakness, infection, hypercalcemia (serum calcium level > 10 mg/dl), and obvious lytic bone lesions on a bone survey, MM was considered a cause of this patient´s intrinsic renal failure. Thus, UPEP, SPEP, and immunoelectrophoresis tests were ordered. We also performed a kidney biopsy. The results of this procedure were known first, and the findings were pathognomonic of myeloma kidney. With the help of bone marrow biopsy and aspirate, we finally concluded that our patient´s AKF was secondary to light chain nephropathy associated with MM [9]. Often, the diagnosis of MM may not be considered in patients with renal insufficiency due to the absence of the other CRAB features, but this case intends to emphasize the importance of recognizing all possible causes of renal failure and thinking about MM as one of them [10]. To the best of our knowledge, acute renal failure as an initial presentation of MM in a young woman, without evidence of hypercalcemia and without lytic bone lesions, remains an uncommon presentation of the disease.

## Conclusions

Renal involvement is a well-documented complication of MM but remains an uncommon sole presentation of the disease. When evaluating the possible causes of acute kidney failure, after excluding pre and post-renal etiologies, MM should be included and pursued as an important possible diagnosis. These patients have poor survival and, therefore, it is of crucial importance to have a high index of suspicion to do an early diagnosis and to promote early and aggressive management. Despite being an invasive procedure, kidney biopsy remains a very significant tool for the differential diagnosis that should not be dismissed.
